# Supplementary material for: A small climate-amplifying effect of climate-carbon cycle feedback
Source: Nat Commun. 2021 May 19;12:2952. doi: 10.1038/s41467-021-22392-w (PMC8134589; doi:10.1038/s41467-021-22392-w)
Supplement: Supplementary file 1 — Supplementary Infomation [file 41467_2021_22392_MOESM1_ESM.pdf]

Supplementary information for

A small climate-amplifying effect of climate-carbon cycle feedback

Xuanze Zhang[1,2*], Ying-Ping Wang[3,4*], Peter J. Rayner[5], Philippe Ciais[6], Kun Huang[2], Yiqi Luo[7], Shilong Piao[8], Zhonglei Wang[9], Jianyang Xia[2], Wei Zhao[10], Xiaogu Zheng[11], Jing Tian[1] and Yongqiang Zhang[1*]

[1] Key Laboratory of Water Cycle and Related Land Surface Processes, Institute of Geographical Sciences and Natural Resources Research, Chinese Academy of Sciences, Beijing 100101, China.

[2] Research Center for Global Change and Ecological Forecasting, School of Ecological and Environmental Science, East China Normal University, Shanghai 200241, China.

[3] Terrestrial Biogeochemistry Group, South China Botanical Garden, Chinese Academy of Sciences, Guangzhou 510650, China.

[4] CSIRO Oceans and Atmosphere, Private Bag 1, Aspendale, Victoria 3195, Australia.

[5] School of Earth Sciences, University of Melbourne, Parkville, Victoria 3010, Australia.

[6] Laboratoire des Sciences du Climat et de l'Environnement, LSCE/IPSL, CEA-CNRS-UVSQ, Université Paris-Saclay, 91191 Gif-sur-Yvette, France.

[7] Center for Ecosystem Science and Society, Northern Arizona University, Arizona, Flagstaff, USA.

[8] Sino-French Institute for Earth System Science, College of Urban and Environmental Sciences, Peking University, Beijing 100871, China.

[9] Wang Yanan Institute for Studies in Economics (WISE) and School of Economics, Xiamen University, Xiamen 361005, China.

[10] National Meteorological Center, China Meteorological Administration, Beijing 100081, China.

[11] Key Laboratory of Regional Climate-Environment Research for East Asia, Institute of Atmospheric Physics, Chinese Academy of Sciences, Beijing 100190, China.

*Correspondence to: Xuanze Zhang (xuanzezhang@igsnrr.ac.cn); Ying-Ping Wang (yingping.wang@csiro.au); Yongqiang Zhang (zhangyq@igsnrr.ac.cn)

Supplementary Information

Supplementary Texts 1-3, Tables 1-4, Figures 1-11, and References.

Text 1. Estimating $\boldsymbol{\beta_k^{BGC}}$ and $\boldsymbol{\gamma_k^{RAD}}$ across different timescales using CMIP5 simulations

We developed a new method for quantifying the two feedback parameters on timescales of Δt years ($\Delta t = 2, \cdots, N-1$), based on the FEA approach, see Supplementary Fig.10. The feedback parameters on the timescale of Δt are defined as the average of all feedback parameters calculated from all time intervals (n) of Δt over the period of N years (e.g. $N =$ 140 years for CMIP5 models): $\beta_k^{BGC} = \frac{1}{n}\sum \beta^{BGC}{}_i$, $\gamma_k^{RAD} = \frac{1}{n}\sum \gamma^{RAD}{}_i$, thus, for $i = 1,2,3, \cdots, n$,

$$\beta_{k=\Delta t}^{BGC} = \frac{1}{n}\sum \frac{\Delta C_{B,i}^{BGC}}{\Delta C_{A,i}^{BGC}} = \frac{1}{n}\sum \frac{C_B^{BGC}(t_0+(i+1)\Delta t) - C_B^{BGC}(t_0+i\Delta t)}{C_A^{BGC}(t_0+(i+1)\Delta t) - C_A^{BGC}(t_0+i\Delta t)} \quad \text{(S1)}$$

$$\gamma_{k=\Delta t}^{RAD} = \frac{1}{n}\sum \frac{\Delta C_{B,i}^{RAD}}{\Delta T_{A,i}^{RAD}} = \frac{1}{n}\sum \frac{C_B^{RAD}(t_0+(i+1)\Delta t) - C_B^{RAD}(t_0+i\Delta t)}{T_A^{RAD}(t_0+(i+1)\Delta t) - T_A^{RAD}(t_0+i\Delta t)} \quad \text{(S2)}$$

where t_0 is the first simulation year of model run, and n is the number of Δt over N simulation years. C_B^{BGC} (in GtC) is the total carbon storage in land and ocean from the BGC simulations, C_B^{RAD} (in GtC) and T_A^{RAD} (in K) are from the RAD simulations. The cumulative airborne fraction on different timescales for the COU simulations (AF_k^{COU}) is

$$AF_{k=\Delta t}^{COU} = \frac{1}{n}\sum \frac{C_A^{COU}(t_0+(i+1)\Delta t) - C_A^{COU}(t_0+i\Delta t)}{C_E(t_0+(i+1)\Delta t) - C_E(t_0+i\Delta t)} \quad \text{(S3)}$$

where the total diagnosed emission is $C_E = \Delta C_A^{COU} + \Delta C_B^{COU}$ from the fully-coupled simulations. According to equation (10), the gain factor over on timescales of Δt years is:

$$g_k^{COU} = 1 - \frac{1}{AF_k^{COU}[1+\beta_k^{BGC}]} \quad \text{(S4)}$$

We estimated the β_k^{BGC} and γ_k^{RAD} across timescales using equations (S3-S4) with the BGC simulations and the RAD simulations with 1% year^{-1} increasing atmospheric CO_2 scenario over 140 years from 9 CMIP5 models (BCC-CSM1-1, CanESM2, CESM1-BGC, HadGEM2-ES, IPSL-CM5A-LR, MIROC-ESM, MPI-ESM-LR, NorESM-ME, UVic ESCM2.9) by Arora et al [1]. The AF_k^{COU} and g_k^{COU} calculated using the fully-coupled (COU) simulations from CMIP5 models. Results are shown in Supplementary Fig.7.

Text 2. Estimating $\boldsymbol{\beta_k^{BGC}}$ and $\boldsymbol{\gamma_k^{COU-BGC}}$ across timescales for C^4MIP coupled/uncoupled simulations

We made use of the coupled and uncoupled simulations of eleven C^4MIP models[2], including the early coupled climate-carbon cycle models of BERN-CC, CSM-1, CLIMBER, FRCGC, HadCM3LC, IPSL-CM2C, LLNL, IPSL-CM4-LOOP, MPI, UMD, UVic-2.7, to estimate β and γ across different timescales. All C^4MIP simulations were forced by anthropogenic fossil fuel emission for 1860-2000 period and by prescribed IPCC SRES A2 CO_2 emission scenario for the 2000-2100 period. Hence, the fully coupled models were emission-driven which calculate the atmospheric CO_2 interactively, with biogeochemically and radiatively coupled in the climate-carbon cycle feedback system. While the "uncoupled" runs in C^4MIP experiments represent that the radiatively coupled process was not active, so that the physical climate would not be affected by CO_2 emission (e.g. non CO_2-induced warming). Following the FEA approach developed by Friedlingstein *et al* [2, 3], we further diagnose the β^{BGC} on timescales of Δt years ($\Delta t = 2, \cdots, N-1$) by

$$\beta_{k=\Delta t}^{BGC} = \frac{1}{n}\sum \frac{C_B^{unc}(t_0+(i+1)\Delta t) - C_B^{unc}(t_0+i\Delta t)}{C_A^{unc}(t_0+(i+1)\Delta t) - C_A^{unc}(t_0+i\Delta t)} \tag{S5}$$

where t_0 is the first simulation year (t_0 =1860) of model run, and n is the number of Δt over $N(= 2100 - 1860)$ simulation years. where C_A^{unc} is the atmospheric CO_2 concentration and C_B^{unc} is the total carbon storage in biosphere (land+ocean), both of which are from the radiatively-uncoupled simulations. The $\gamma^{COU-BGC}$ on timescales from the fully-coupled and uncoupled simulation of each C^4MIP model are calculated by,

$$\gamma_{k=\Delta t}^{COU-BGC} = \frac{1}{n}\sum \frac{[C_B^{cou}(t_0+(i+1)\Delta t) - C_B^{cou}(t_0+i\Delta t)] - [C_B^{unc}(t_0+(i+1)\Delta t) - C_B^{unc}(t_0+i\Delta t)]}{T_A^{cou}(t_0+(i+1)\Delta t) - T_A^{cou}(t_0+i\Delta t)} \tag{S6}$$

where C_A^{cou}, C_B^{cou} and T_A^{cou} are from the fully-coupled simulations. We also estimate the airborne fraction (*AF*) and the gain factor (g) on timescales for each C^4MIP model using Equations (S3-4) in the Methods section. Results are shown in Supplementary Fig.8.

Text 3. Comparison of the nonlinear feedback contributions estimated in this study with previous modeling studies

In this study, we defined the nonlinear feedback term as: $f(\beta,\gamma) = [\Delta C_B^{COU} - (\Delta C_B^{BGC} + \Delta C_B^{RAD})]/\Delta C_A^{COU}\Delta T_A^{COU}$ and estimated its contributions to the feedback parameters β and γ* (in units of GtC ppm^{-1} or GtC K^{-1}), whereas previous studies (i.e., Gregory et al., 2009; Schwinger et al., 2014)[4, 5] only estimated the nonlinear feedback contributions to the overall carbon uptakes by land and ocean or by ocean only (in units of Gt C) based on model simulations. Schwinger et al. (2014) estimated that the contribution of nonlinearity to the estimated carbon uptake by ocean under fully coupled simulation was relatively small (3.6% to 10.6%). Our results show that the nonlinear feedback ($f(\beta,\gamma)\Delta C_A$) only has very small impact (3±3%) on the estimated carbon-concentration feedback parameter (β of land + ocean), and that the contribution of nonlinear feedback to the climate-carbon feedback parameter (γ^* or $\gamma^{COU-BGC}$ of land + ocean) is 15±23% across the CMIP5 models (see Supplementary Table 4).

We further estimated the nonlinear contributions to feedback parameters with $f(\beta,\gamma)$ defined in this study using the published results from Gregory et al., 2009 or Schwinger et al. (2014). We showed that using the modeling result in Schwinger et al. (2014)'s study, the non-linear contribution to the ocean γ-feedback γ_O^{nl} is -9.9 GtC K^{-1}, which is about 60% of the total ocean γ-feedback $\gamma_O^{COU-BGC}$ (-16.6 GtC K^{-1}). The non-linear contribution to the ocean γ-feedback β_O^{nl} is -0.053 GtC ppm^{-1}, which is only 6.6% of the $\beta_O^{COU-BGC}$ (0.801 GtC ppm^{-1}).

Moreover, by using the result of the HadCM3LC model under the 1% yr^{-1} CO_2 increase scenario from Fig.3 in Gregory et al. (2009), we estimated that the non-linear contribution to the land+ocean γ-feedback is -53.84 GtC K^{-1} which is 45% of the total land+ocean γ-feedback $\gamma_B^{COU-BGC}$ (-119 GtC K^{-1}), and the non-linear contribution to the land+ocean β-feedback is -0.33 GtC ppm^{-1}, which is 20% of the land+ocean β-feedback $\beta_B^{COU-BGC}$ (1.65 GtC ppm^{-1}).

These estimations based on Gregory et al. (2009) are noticeably larger than our results from the nine CMIP5 models (Supplementary Table 4). For example, for the HadGEM2 in CMIP5 models, the largest non-linear contribution to γ is -28.71 GtC K^{-1} (45% of the total γ-feedback) and the largest non-linear contribution to β is -0.185 GtC ppm^{-1} (9.6% of the total β -feedback). However, across CMIP5 models, the non-linear contributions show large spreads (0.2 to 9.6% for β-feedback and 0.8 to 45% for γ-feedback, see Supplementary Table 4), implying that there exist large uncertainties of the nonlinearity in CMIP5 models and the HadCM3LC used in Gregory et al. (2009).

Supplementary Table 1 | Estimates of β from observational datasets over 1880-2017.

Observational Dataset	***p*** (ppm ppm^{-1})	***β*** (GtC ppm^{-1})
**HadCRUT4**	2.5088 ±0.3037	3.199 ±0.644
**Berkeley Earth**	2.5024 ±0.3030	3.185 ±0.642
**GISTEMP**	2.5233 ±0.3059	3.229 ±0.648
**NOAAGlobalTemp**	2.5362 ±0.3078	3.257 ±0.652
**Ensemble Mean**	**2.5177**	**3.217**
**+/-S.D.**	**±0.153**	**±0.323**

Supplementary Table 2 | Estimates of γ_{100yr} from temperature reconstructions and CO_2 ice-core records over 1000-1850 with optimal β=3.22±0.32 GtC ppm^{-1} (Supplementary Table 1).

Ice-core CO_2 records	Reconstructed Temperature	η_{100yr} (ppm °C^{-1})	γ_{100yr} (GtC °C^{-1})
**Low Dome**	PAGES2k	45.35	-242.06
	Frank2010	20.75	-110.74
	Mann2009	32.92	-175.76
	Mann EIV	23.14	-123.51
	Moberg2005	10.54	-56.24
**WAIS**	PAGES2k	28.91	-154.3
	Frank2010	13.23	-70.59
	Mann2009	20.99	-112.04
	Mann EIV	14.75	-78.73
	Moberg2005	6.72	-35.85
**DML**	PAGES2k	43.7	-233.24
	Frank2010	19.99	-106.7
	Mann2009	31.73	-169.36
	Mann EIV	22.3	-119.01
	Moberg2005	10.15	-54.19
**Ensemble Mean**		**23.01**	**-122.82**
**+/-S.D.**		**+/-11.27**	**+/-60.16**

Supplementary Table 3 | Estimates of β and γ for eleven C^4MIP models for the periods of 1880-2017 and 1880-2100.

Model	β^{BGC} (GtC ppm^{-1})		$\gamma^{COU-BGC}$ (GtC K^{-1})	
	**1880-2017**	**1880-2100**	**1880-2017**	**1880-2100**
**BERN-CC**	3.52	2.94	-38.97	-61.23
**CCSM-1**	2.46	1.97	-12.97	-21.77
**CLIMBER**	2.47	1.99	-17.99	-41.21
**FRCGC**	3.18	2.36	-36.29	-72.19
**HadCM3LC**	2.95	2.14	-41.19	-113.29
**IPSL-CM2C**	3.49	3.25	-13.22	-48.79
**IPSL-CM4-LOOP**	3.17	2.34	-10.20	-19.05
**LLNL**	4.27	3.70	-25.89	-32.71
**MPI**	3.27	2.50	-36.83	-44.11
**UMD**	1.67	1.71	-30.64	-52.22
**UVic-2.7**	3.26	2.29	-38.54	-67.40
**Ensemble Mean**	**3.07**	**2.47**	**-27.52**	**-52.18**
**±S.D.**	**±0.68**	**±0.60**	**±11.93**	**±26.54**

Supplementary Table 4 | Estimates of $\boldsymbol{\beta}, \boldsymbol{\gamma}$ and $\boldsymbol{f(\beta,\gamma)}$ for the nine CMIP5 models from the $1pctCO_2$ climate-carbon cycle feedback experiments.

Model	β^{BGC}	$f(\beta,\gamma)\Delta T_A$	$\gamma^{COU-BGC}$	γ^{RAD}	$f(\beta,\gamma)\Delta C_A$	$f(\beta,\gamma)$
	(GtC ppm^{-1})	(GtC ppm^{-1})	(GtC K^{-1})	(GtC K^{-1})	(GtC K^{-1})	(GtC ppm^{-1} K^{-1})
**BCC-CSM1**	2.06	-0.045	-89.95	-86.22	-9.71	-11.35×10^{-3}
**CanESM2**	1.66	0.004	-75.75	-78.82	0.64	0.75×10^{-3}
**CESM1-BGC**	0.96	0.022	-17.27	-23.77	5.04	5.89×10^{-3}
**HadGEM2**	1.92	-0.185	-63.34	-44.04	-28.71	-33.57×10^{-3}
**IPSL-CM5A-LR**	2.05	-0.036	-63.06	-65.13	-6.08	-7.15×10^{-3}
**MIROC-ESM**	1.55	-0.041	-104.15	-100.14	-6.3	-7.41×10^{-3}
**MPI-ESM-LR**	2.30	-0.111	-102.38	-88.09	-18.98	-22.19×10^{-3}
**NorESM-ME**	1.11	-0.042	-21.69	-14.08	-9.76	-11.41×10^{-3}
**UVic ESCM2.9**	1.74	-0.058	-93.67	-85.44	-12.59	-14.60×10^{-3}
**Ensemble Mean**	**1.71**	**-0.055**	**-70.14**	**-65.08**	**-9.6**	$\mathbf{-11.22\times10^{-3}}$
**±S.D.**	**±0.44**	**0.062**	**±32.43**	**±30.74**	**±10.03**	$\mathbf{\pm11.72\times10^{-3}}$

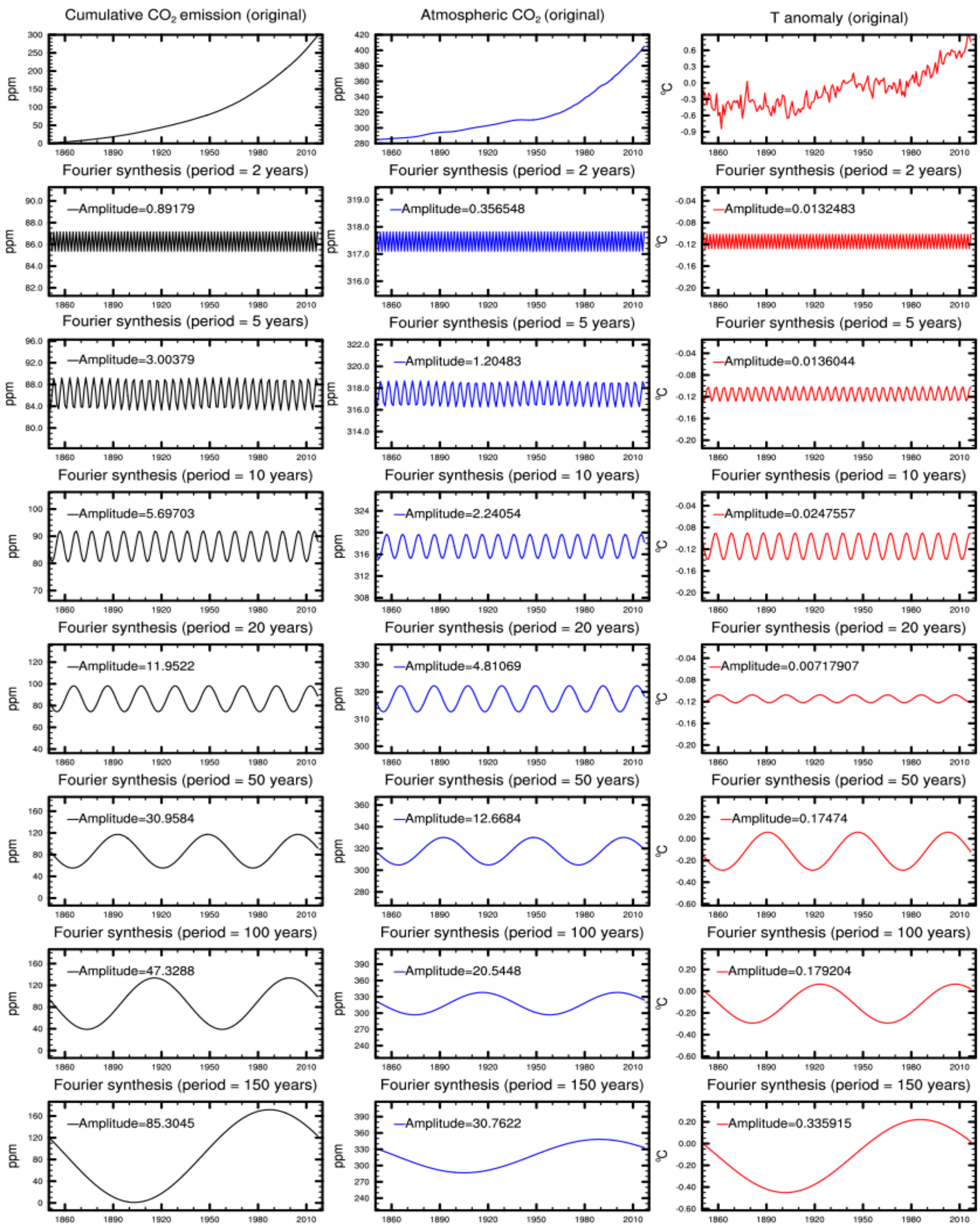

Supplementary Fig. 1 | Fourier analysis of historical anthropogenic cumulative CO_2 emission, atmospheric CO_2, and global mean temperature anomaly over 1850-2017. Left panels, Fourier synthesis time series of cumulative CO_2 emission mean (C_E) at periods of 2, 5, 10, 20, 50, 100, 150 years, respectively. **Middle panels,** Fourier synthesis time series of atmospheric CO_2 (C_A) at periods of 2, 5, 10, 20, 50, 100, 150 years, respectively. **Right panels,** Fourier synthesis time series of global mean temperature (T_A) from HadCRUT4 at periods of 2, 5, 10, 20, 50, 100, 150 years, respectively. Amplitudes for C_E, C_A, and T_A at all timescales (k) were used for estimating ${TCRE^{-1}}_k$ and ${\alpha^{-1}}_k$ in this study (Methods).

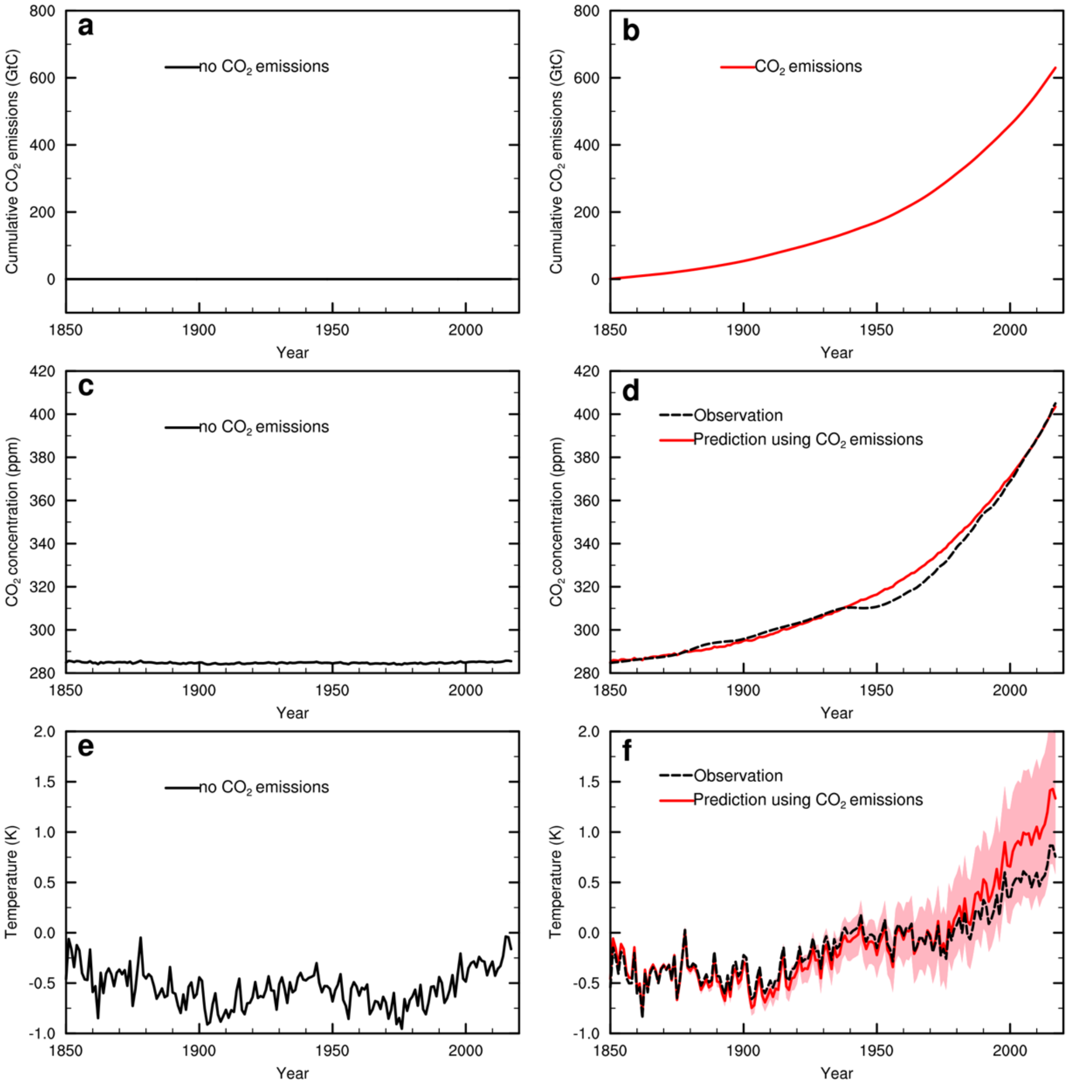

Supplementary Fig. 2 | Simulations of CO_2 and temperature using a simple three parameters box model for climate-carbon cycle feedback analysis. The box model (see equations (32-33) in Method) was used here to predict temperature and CO_2 over 1850-2017 by setting β=3.22 GtC ppm^{-1}, γ^*=-10.9 GtC K^{-1}, s =3±1.5 K and ε being the detrended T_A anomaly time series from HadCRUT4 using annual cumulated CO_2 emissions as input (**b, d, f**) or no CO_2 emissions input (**a, c, e**). Result shows predicted atmospheric CO_2 driven by CO_2 emissions is very close to the observation ($R^2 = 0.99, RMSE = 3.5\,ppm$) (**d**), but predicted temperature shows larger increasing trend from 1980-2017 ($R^2 = 0.96, RMSE = 0.17\,K$) (**f**). The shaded area in plots are the uncertainty due to s varied from 1.5 to 4.5 K.

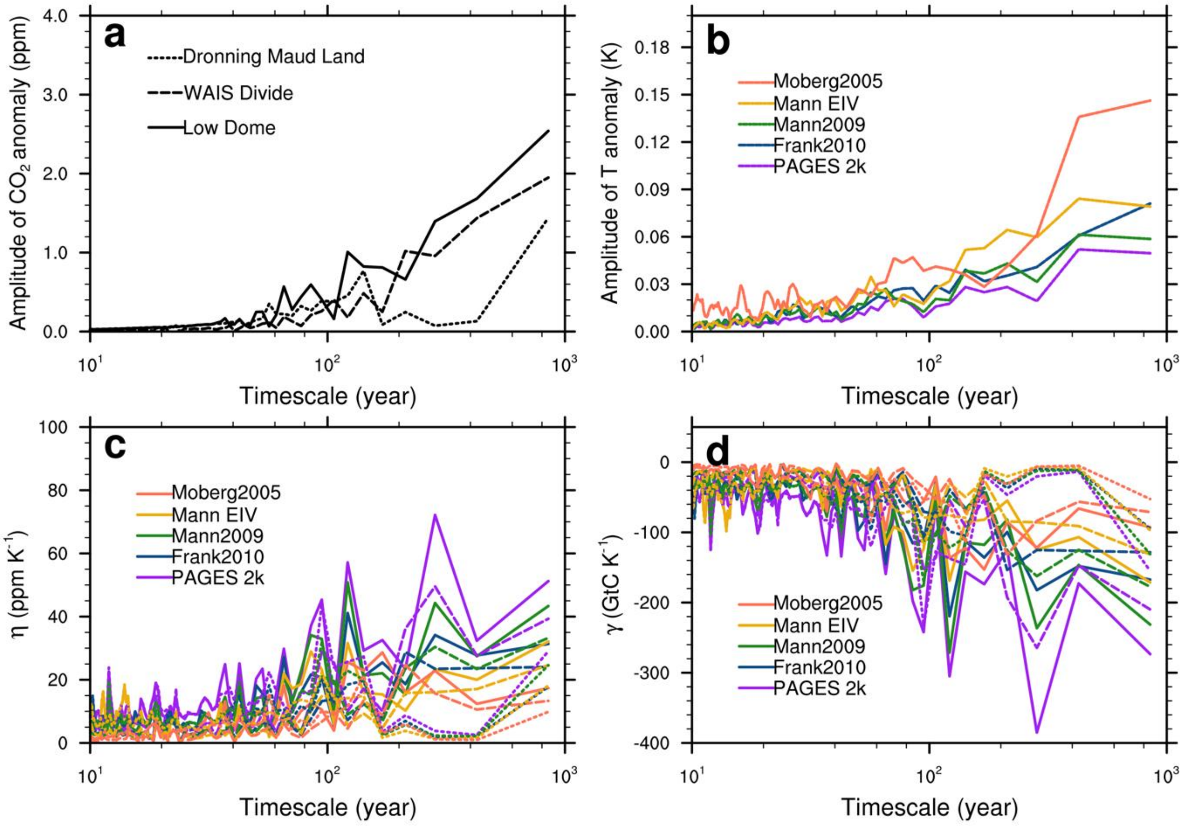

Supplementary Fig. 3 | Climate variability and feedback across timescales for the period 1000-1850. a, Amplitude spectrum for atmospheric CO_2 from three Antarctic ice-core CO_2 records (Low Dome, WAIS Divide and Dronning Maud Land) using Fourier analysis. **b,** Same as a but for hemispheric mean temperature from 5 reconstructions (PAGES 2k, Frank2010, Mann2009, Mann EIV, Moberg2005). **c,** Ensemble estimates of η across timescales from 15 combinations of 3 ice-core CO_2 records × 5 reconstructed temperature reconstructions. **d,** Same as c but for the climate change feedback parameter γ.

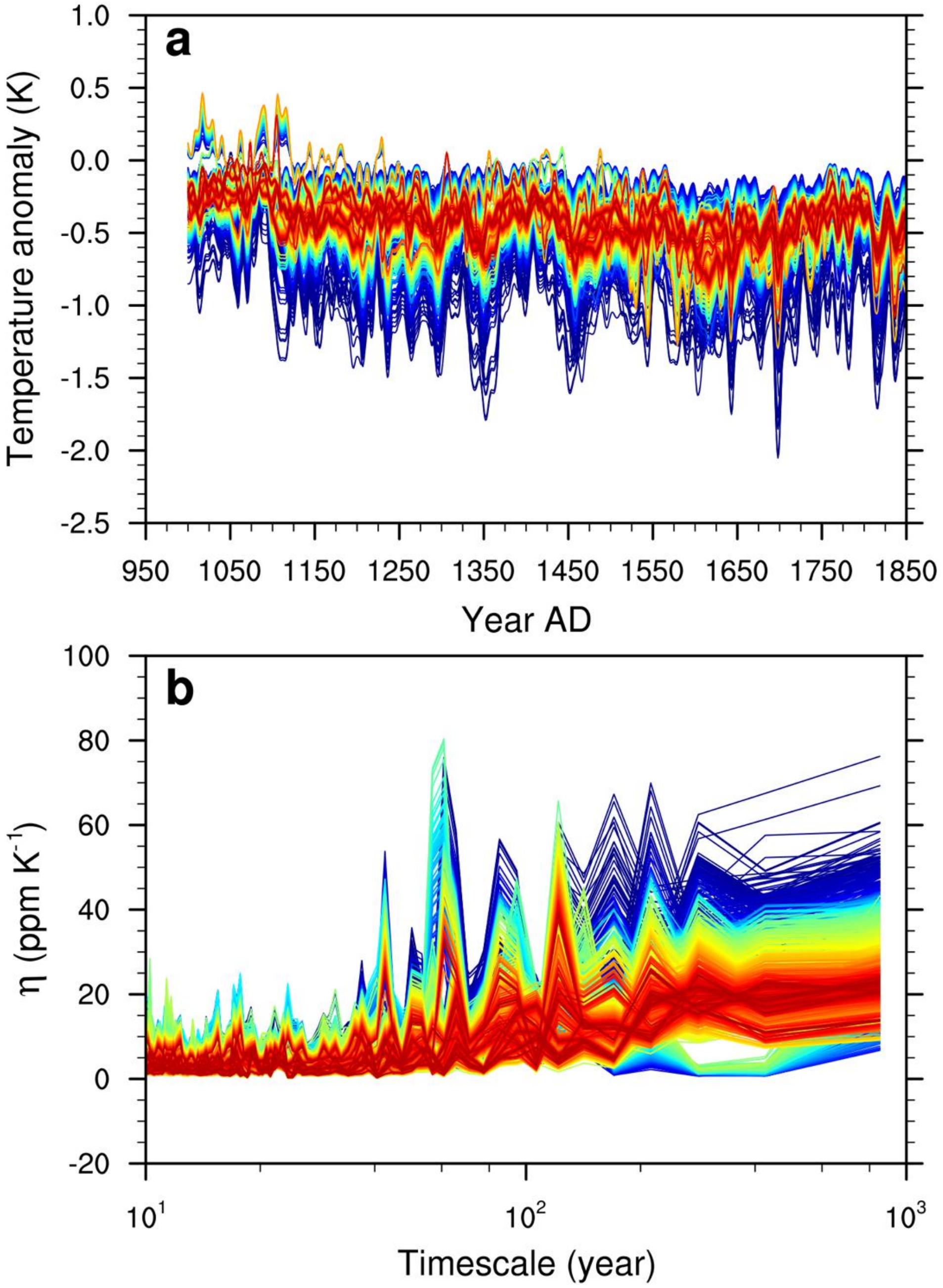

Supplementary Fig. 4 | Large ensemble estimates of temperature variability and climate-carbon cycle feedback. a, 521 estimates of calibrated temperature anomaly reconstructions over the period 1000-1850 from Frank et al. 2010. **b,** Ensemble estimates of η across timescales from >1,500 members (based on 3 ice-core CO_2 records × 521 calibrated temperature reconstructions, EnOBS) over the period 1000-1850. Coloring of encumber members in **a** and **b** are based on distance from their ensemble mean.

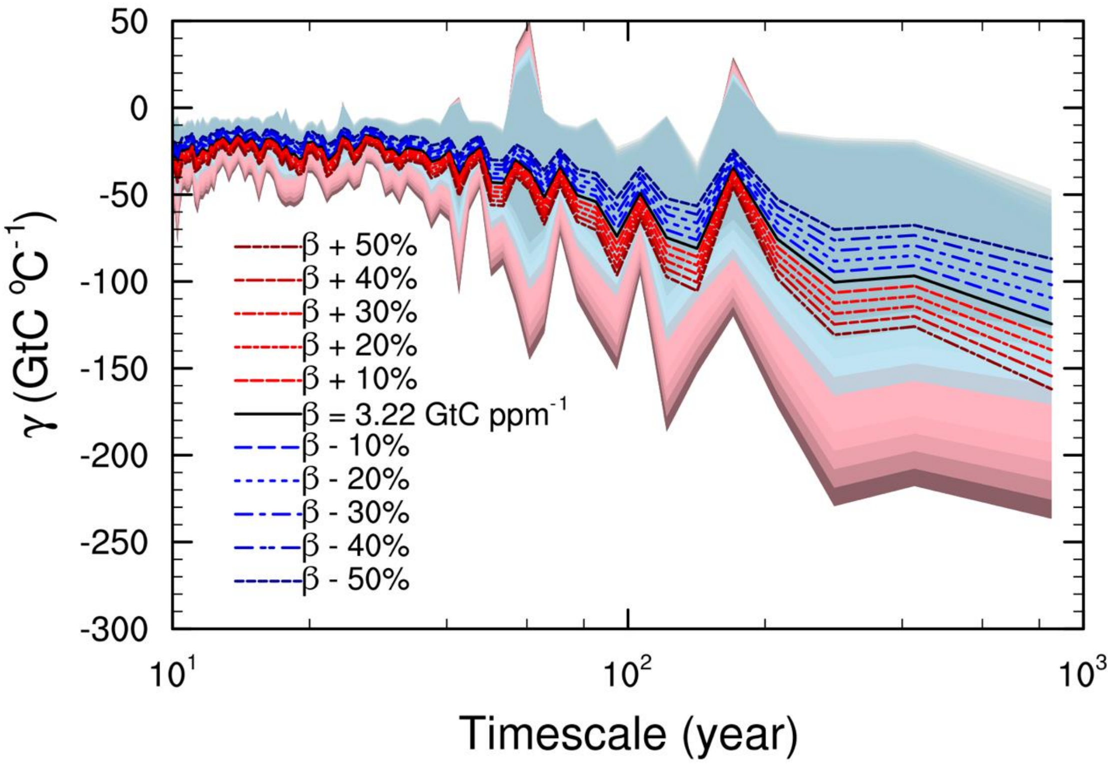

Supplementary Fig. 5 | Error sensitivity analysis of γ on different timescales in response to changes in β. The β is 3.22 GtC ppm^{-1} with an uncertainty of 10%, estimated from observational datasets over 1880-2017. We applied this value of β to estimate pre-industrial γ on timescales from 10 to 1000 years using the EnOBS ensemble η across timescales from >1,500 members (based on 3 ice-core CO_2 records ×521 calibrated temperature reconstructions [6]). This figure shows that when the β has overestimations (or underestimations) of 10% to 50% of its industrial observation-based value (3.22 GtC ppm^{-1}), the γ shows underestimations (or overestimations) of about 6% to 30%. This largest change of 30% (~25 GtC K^{-1}) in γ_{100yr} is still smaller than the uncertainty of γ_{100yr} (a standard deviation of 41.90 GtC K^{-1}) that was mainly caused by the large divergences in the three ice-core CO_2 records and reconstructed temperatures.

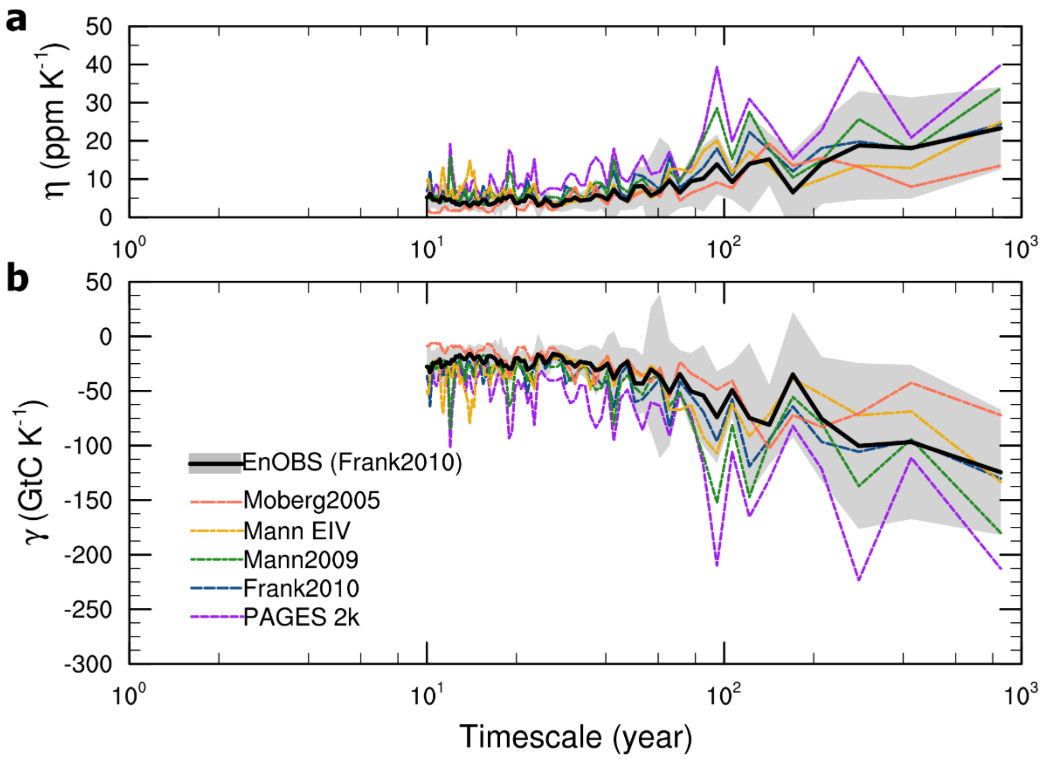

Supplementary Fig. 6 | Ensemble estimates of climate-carbon feedback parameters for the period 1000-1850. **a**, Evolution of the η estimates across timescales of 10-850 years derived from 5 reconstructed temperature members (on average over 3 ice-core CO_2 record combinations) and from EnOBS ensemble mean with uncertainty (±1σ) of >1,500 members (3 ice-core CO_2 × 521 calibrated temperature reconstructions) for 1000-1850. **b**, Same as **a**, but for evolution of the γ estimates across timescales of 10-850 years.

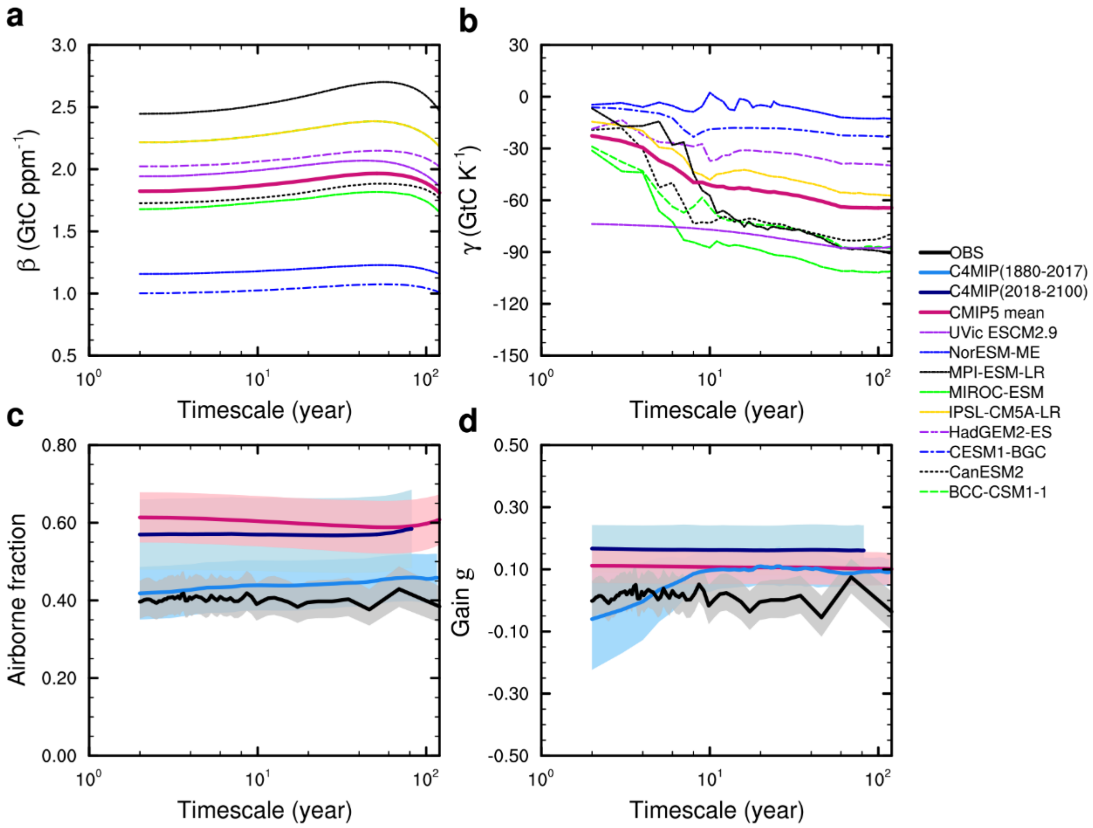

Supplementary Fig. 7 | Estimates of the climate-carbon cycle feedback parameters over timescales derived from the CMIP5 and C⁴MIP models. **a**, Evolution of the β^{GBC} estimates across timescales derived from the biogeochemically coupled simulations of nine CMIP5 models. **b**, Same as **a** but for the γ^{RAD} estimates from the radiatively coupled simulations. **c**, Evolution of the cumulative airborne fraction (AF) estimates (mean ±1σ) across timescales from observations and from the fully coupled simulations of the CMIP5 and the C⁴MIP models. **d**, Same as **c** but for the feedback gain factor (g) derived from the β and AF estimates. Observation-based estimates were calculated for the period 1880-2017. All CMIP5 model simulations were forced by 1% year^{-1} increasing atmospheric CO_2 concentrations over 140 years with an initial level at 285 ppm [1]. All C⁴MIP models were forced by the historical and the prescribed IPCC SRES A2 CO_2 emissions scenario for the 1860-2100 period.

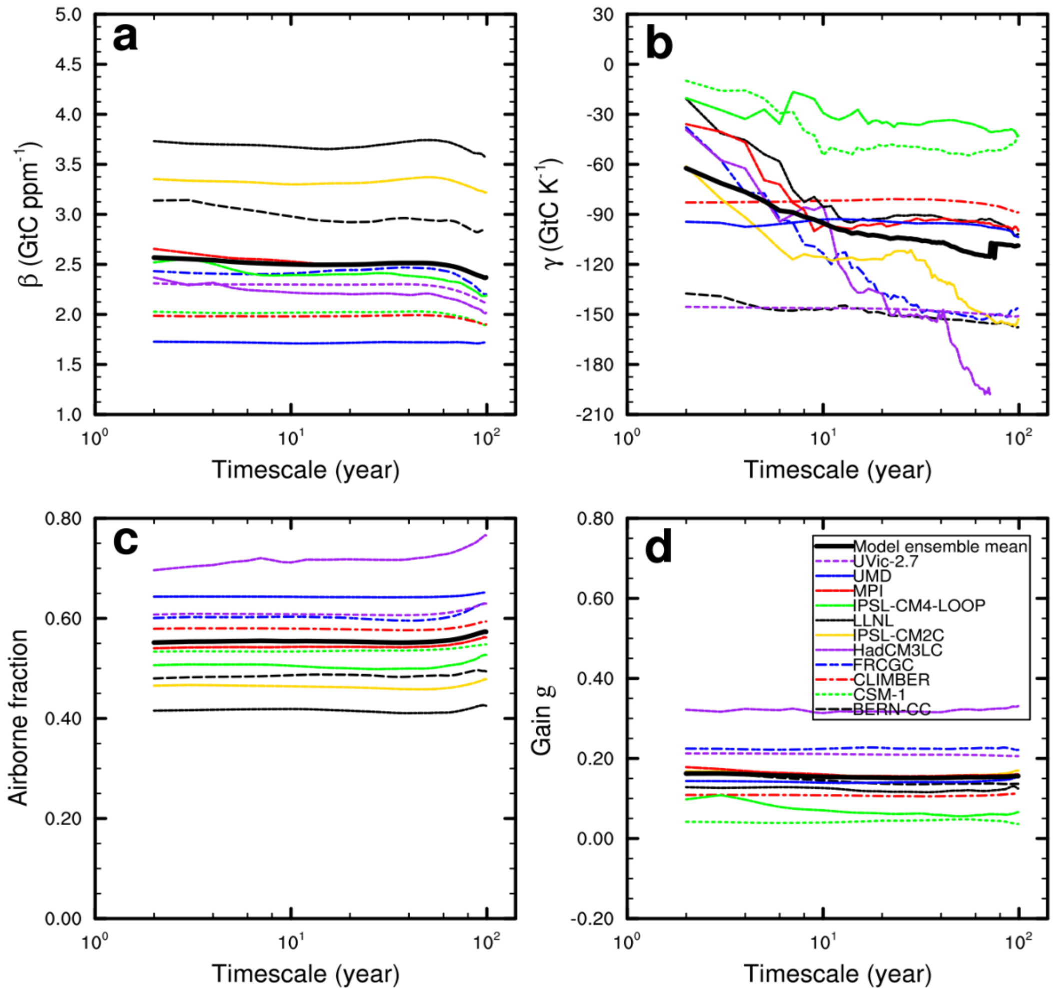

Supplementary Fig. 8 | Estimates of the climate-carbon cycle feedback parameters over timescales from the coupled/uncoupled simulations of eleven C⁴MIP models for the period 1880-2100. a, Evolution of the β^{BGC} estimates across timescales from the uncoupled simulations with equation (S1). **b**, Evolution of the $\gamma^{COU-BGC}$ estimates across timescales from the fully-coupled simulations with equation (S2). **c**, Evolution of the airborne fraction (AF) estimates across timescales from the fully-coupled simulations. **d**, Evolution of the feedback gain factor (g) estimates across timescales from the β and AF estimates. All C⁴MIP models simulations were forced by anthropogenic fossil fuel CO_2 emissions for the historical and the prescribed IPCC SRES A2 CO_2 emissions scenario for the 1860-2100 period.

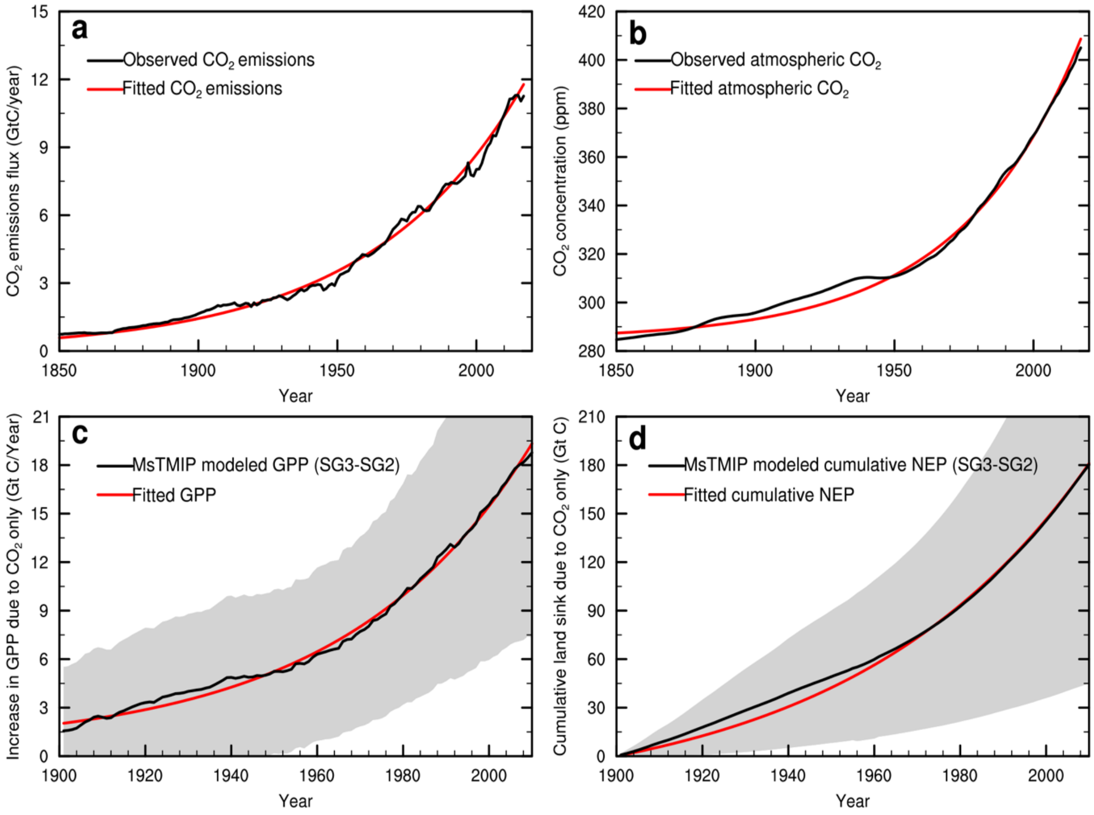

Supplementary Fig. 9 | Exponential growths of global annual CO_2 emissions flux, atmospheric CO_2 concentration, GPP flux, and cumulative land carbon sink during industrial period. a, Comparison of observed and fitted CO_2 emissions (Gt C year^{-1}) over 1850-2017. The fitted CO_2 emissions (in red) was calculated by $y = 0.27e^{0.018t}$. **b,** Comparison of observed and fitted atmospheric CO_2 (ppm) over 1850-2017. The fitted atmospheric CO_2 (in red) was calculated by $y = 2.6e^{0.023t} + 285$. **c,** Comparison of modeled ensemble mean GPP and fitted GPP increase (Gt C year^{-1}) over 1901-2010. The fitted GPP increase (in red) was calculated by $y = 1.5e^{0.023t} + 0.5$. **d,** Comparison of modeled ensemble mean cumulative NEP and fitted cumulative NEP (Gt C) over 1901-2010. The fitted cumulative NEP (in red) was calculated by $y = 29e^{0.018t} - 29$. The modeled GPP and NEP were calculated from the differences between experiment SG3 and experiment SG2 of an ensemble of 14 MsTMIP terrestrial ecosystem models. The shaded area in **d** and **c** represents an uncertainty of one standard deviation (in grey-shaded) in MsTMIP ensemble simulations. In the experiment SG2 of the MsTMIP project [7], all 14 terrestrial ecosystem models were forced by historical observational climate and land use change data over 1901-2010 and a constant CO_2 value of 1901 over 1901-2010. In the experiment SG3, all 14 MsTMIP models were forced by historical observational climate, land use change data, and annual CO_2 values over 1901-2010. The difference of SG3-SG2 represents that the MsTMIP models were driven only by increasing atmospheric CO_2 over 1901-2010.

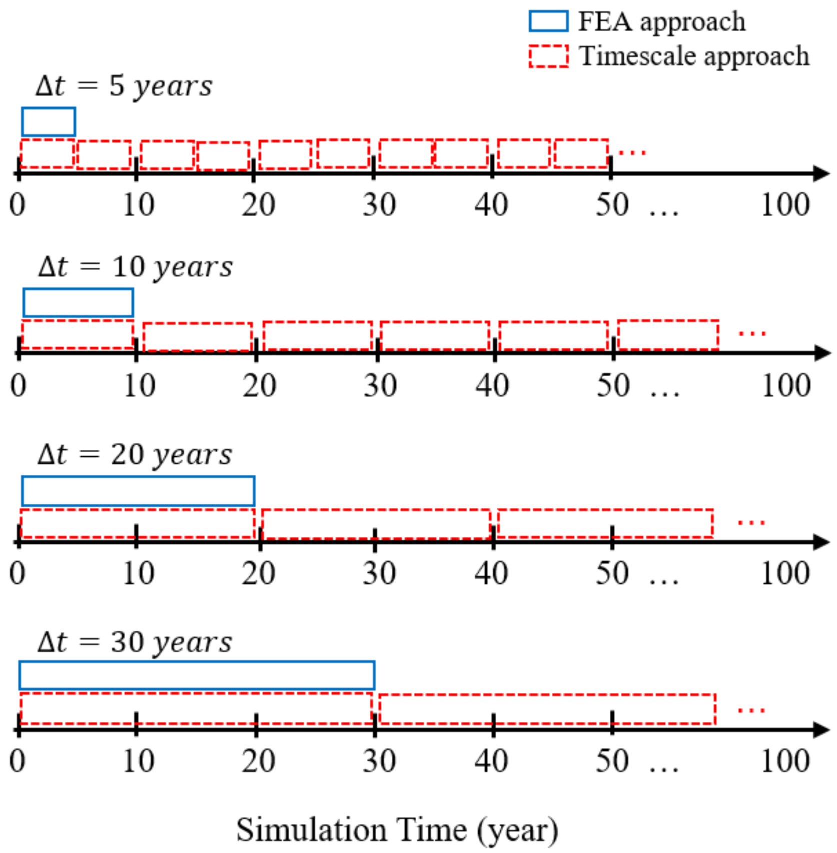

Supplementary Fig. 10 | A diagram to show the difference between the FEA approach and the timescale approach used in this study. The FEA approach is used to calculate β^{BGC} and γ^{RAD} for different time intervals since the first simulation year (boxes in blue for different Δt intervals), and the timescale approach is used to calculate β_k^{BGC} and γ_k^{RAD} on different time scales (boxes in red for different Δt intervals), which are the average of all β^{BGC} and γ^{RAD} from all time intervals (n) of Δt over the period of N years. See also equations (S1-S2), more details about the timescale approach is shown in Texts 1-2.

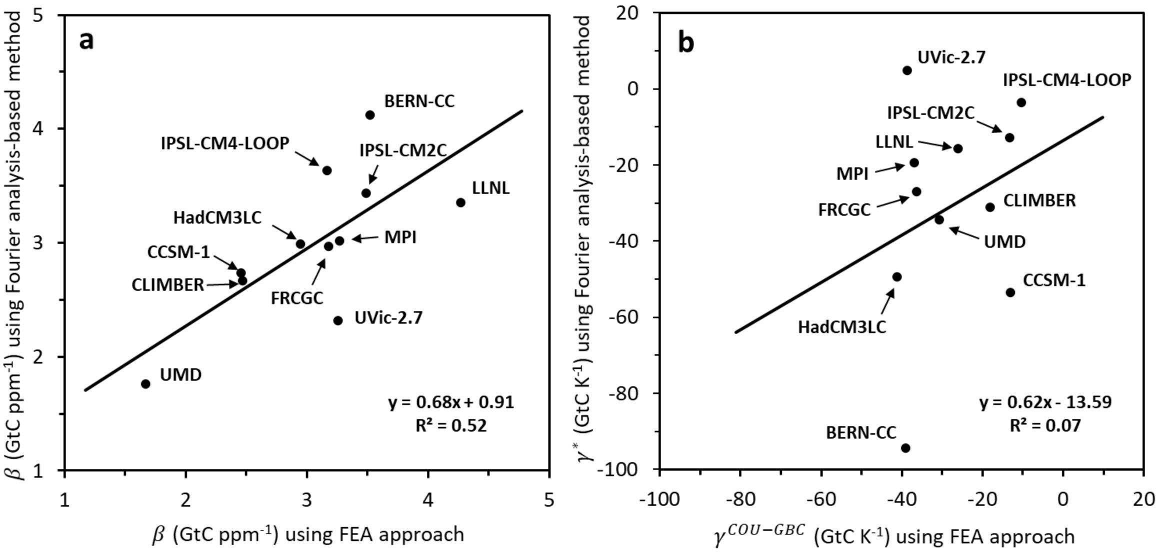

Supplementary Fig. 11 | Comparison of β and γ^* during 1880-2017 for the C[4]MIP models from between Fourier analysis-based method and the FEA-based approach. The historical observed CO_2 emissions, and global atmospheric CO_2 concentration, global mean near-surface temperature, global land and ocean carbon uptakes derived from the COU (fully-coupled) simulations of 11 C[4]MIP models were used. Our result showed that the β for the C[4]MIP ensemble from the Fourier analysis-based method is 2.997±0.556 GtC ppm^{-1}, which is close to the value of 3.064±0.680 GtC ppm^{-1} for the C[4]MIP from the FEA approach. The γ^* for the C[4]MIP ensemble estimated using the Fourier analysis-based method is -30.66 ± 18.72 GtC K^{-1}, which is about 10% larger in magnitude than the FEA approach-based estimate (-27.52±11.92 GtC K^{-1}). Then, the Fourier analysis-based g for the C[4]MIP using equation (10) is 0.09±0.05, which is consistent with the FEA approach-based g (0.09±0.04).

References

1. Arora, V. K., *et al.* Carbon–Concentration and Carbon–Climate Feedbacks in CMIP5 Earth System Models. *J. Clim.* **26**, 5289-5314 (2013).

2. Friedlingstein, P., *et al.* Climate–Carbon Cycle Feedback Analysis: Results from the C4MIP Model Intercomparison. *J. Clim.* **19**, 3337-3353 (2006).

3. Friedlingstein, P., Dufresne, J. L., Cox, P. M. & Rayner, P. How positive is the feedback between climate change and the carbon cycle? *Tellus B* **55**, 692-700 (2003).

4. Gregory, J. M., Jones, C. D., Cadule, P. & Friedlingstein, P. Quantifying Carbon Cycle Feedbacks. *J. Clim.* **22**, 5232-5250 (2009).

5. Schwinger, J., *et al.* Nonlinearity of Ocean Carbon Cycle Feedbacks in CMIP5 Earth System Models. *J. Clim.* **27**, 3869-3888 (2014).

6. Frank, D. C., *et al.* Ensemble reconstruction constraints on the global carbon cycle sensitivity to climate. *Nature* **463**, 527-530 (2010).

7. Huntzinger, D. N., *et al.* Uncertainty in the response of terrestrial carbon sink to environmental drivers undermines carbon-climate feedback predictions. *Scientific reports* **7**, 4765 (2017).
